# Diagnostic Role of Shear Wave Elastography for Differentiating Benign and Malignant Breast Lesions With Histopathological Examination (HPE) Correlation

**DOI:** 10.7759/cureus.104103

**Published:** 2026-02-23

**Authors:** Himangshu Rajbongshi, Mousumi Gogoi, Sukanya Goswami, Bijit K Duara

**Affiliations:** 1 Department of Radiology, Gauhati Medical College and Hospital, Guwahati, IND

**Keywords:** b-mode ultrasonography, breast cancer, invasive ductal cell carcinoma, shear wave elastography, tissue stiffness

## Abstract

Breast cancer is a leading cause of mortality worldwide, with invasive ductal carcinoma being the most common malignant tumor. Ultrasonography (USG) is the initial imaging modality for breast lesions, but its low specificity often necessitates invasive histopathological examination (HPE). Shear wave elastography (SWE) is a novel technique that assesses tissue stiffness non-invasively, providing promising results in differentiating benign and malignant breast lesions. This study evaluates the diagnostic accuracy of SWE combined with B-mode USG in characterizing breast lesions and correlates findings with HPE results. A hospital-based prospective observational study was conducted on 50 female patients aged 18 years and above with breast lesions categorized as Breast Imaging-Reporting and Data System (BI-RADS) 3 or higher. SWE demonstrated a sensitivity of 72.72%, a specificity of 85.71%, and a diagnostic accuracy of 80%, highlighting its potential to reduce unnecessary biopsies. This study concludes that SWE is a valuable adjunct to conventional USG for breast lesion evaluation.

## Introduction

Breast cancer is one of the leading causes of mortality and morbidity worldwide, with a wide spectrum of pathologies ranging from benign to malignant lesions. Fibroadenoma is the most common benign breast lesion with an overall incidence of about 2.2% [1], while invasive ductal carcinoma is the most frequently diagnosed malignant tumor, representing about 80% of all diagnoses [2]. Breast cancer accounts for 13.5% of new cancer cases and 10.6% of all cancer deaths in India, making it the country's largest cause of cancer incidence and mortality, according to the Global Cancer Observatory (GCO) [3]. Despite the implementation of the National Programme for Non-Communicable Diseases (NP-NCD), which includes breast cancer screening for women aged 30-69 years, only 1.6% of women in this age group have undergone screening [4]. This low screening rate contributes to late-stage diagnosis, which is a common concern in India.

For risk assessment and quality control in mammography, ultrasonography (USG), and magnetic resonance imaging (MRI), the Breast Imaging-Reporting and Data System (BI-RADS) is extensively utilized [5]. BI-RADS categorizes breast lesions into six categories, ranging from benign (BI-RADS 1 and 2) to malignant (BI-RADS 5 and 6). While B-mode USG is the preferred initial imaging modality for differentiating solid and cystic breast lesions, its low specificity often necessitates invasive histopathological examination (HPE), which remains the gold standard for diagnosis. However, HPE is invasive and can lead to complications, making non-invasive diagnostic methods highly desirable. ​

Breast cancer incidence in India shows a notable disparity between urban and rural populations, with urban areas experiencing higher rates attributed to lifestyle, dietary, and reproductive factors. Key risk factors include nulliparity, early menarche, multiple abortions, high-fat intake, genetic mutations (BRCA1 and BRCA2), and lifestyle choices such as alcohol use and obesity. Early detection is vital for survival, with imaging options including mammography, MRI, and USG. Mammography is effective for women 40 and older, wherein digital tomosynthesis enhances mass detection in dense tissue. Ductography is useful for assessing intraductal tumors, and high-resolution USG is beneficial for younger women with dense breasts, often serving as the first imaging approach for palpable lumps.

USG elastography has emerged as a promising technique for the diagnosis of malignant breast tumors [6]. It assesses tissue elasticity or stiffness, which is inversely correlated with the deformability of the tissue. There are two main types of elastography: strain elastography and shear wave elastography (SWE). Strain elastography uses manual compression to measure tissue deformation, providing qualitative data. SWE, on the other hand, uses acoustic radiation force impulse (ARFI) to generate pressure waves that propagate transversely, measuring tissue stiffness quantitatively in kilopascals. Studies have shown that combining elastography with B-mode USG significantly improves diagnostic accuracy [7,8]. ​

The Tsukuba elasticity scoring system is widely used in elastography to evaluate breast lesions [9]. It assigns scores from 1 to 5 based on the stiffness and elasticity of the tissue, with higher scores indicating a higher likelihood of malignancy. Studies have shown that elastography offers a high sensitivity and specificity for distinguishing between benign and malignant breast lesions. For example, studies by Thomas et al. and Lee et al. have shown that combining elastography with B-mode USG improves the positive predictive value and diagnostic accuracy of breast lesion evaluation [10,11]. ​

Malignant breast lesions typically originate from the terminal duct lobular unit (TDLU) and can be classified into carcinomas and sarcomas. Carcinomas, which arise from epithelial cells, are the most common type of breast cancer. Ductal carcinoma in situ (DCIS) and lobular carcinoma in situ (LCIS) are examples of preinvasive lesions that can develop into invasive ductal carcinoma and invasive lobular carcinoma, respectively [12]. Phyllodes tumors, Paget's disease of the nipple, and inflammatory breast cancer are other forms of breast cancer. Tumor size, involvement of axillary lymph nodes, histological grade, and hormone receptor status are all prognostic markers for breast cancer. ​

The gold standard for breast cancer diagnosis remains histopathological examination, which can be performed using fine needle aspiration biopsy (FNAC), core needle biopsy, or surgical biopsy [13]. However, these methods are invasive and may lead to complications. Non-invasive imaging modalities like SWE offer a promising alternative for breast lesion evaluation, potentially reducing the need for biopsies and improving patient outcomes. ​

With an emphasis on SWE's sensitivity, specificity, and diagnostic accuracy, this study attempts to assess the diagnostic utility of SWE in distinguishing between benign and malignant breast lesions. By correlating SWE findings with HPE results, the study seeks to establish SWE as a reliable and efficient tool for breast cancer diagnosis.

## Materials and methods

Study design

This is a hospital-based prospective observational study conducted in the Department of Radiodiagnosis, Gauhati Medical College and Hospital, Guwahati, India, with due approval from the institute's Institutional Ethics Committee (approval number: 190/2007/Oct.2022/49; date: 23/12/2022). The duration of the study was 12 months, spanning from October 2022 to September 2023.

Study subjects

The study subjects comprised females aged 18 years and above presenting with breast lesions being referred to the Department of Radiodiagnosis, Gauhati Medical College and Hospital, for the evaluation of breast lesions.

Inclusion and exclusion criteria

Included were females aged 18 years and above with breast lesions categorized as BI-RADS 3 or higher and lesions with a size of >5 mm. In contrast, postoperative breast lesions or breast lesions initially assessed as BI-RADS 1 and 2 were excluded from the study.

The BI-RADS category was classified as follows: BI-RADS 1: normal; BI-RADS 2: benign (absence of any suspicious finding plus simple cyst and uniform and intense hyperechogenicity); BI-RADS 3: probably benign; BI-RADS 4: suspicious abnormality (one or more suspicious findings); and BI-RADS 5: highly suggestive of malignancy (two or more major suspicious findings) [5].

Sample size

The study's sample size was 50, calculated using the formula $n=\frac{Z^{2}P(1-P)}{d^{2}}$ where Z is equal to 1.96 for a confidence level of 95%, P is the prevalence (expressed as a decimal) of 5%, and d is the precision of error (0.1386).

Methodology

After obtaining informed consent, patients underwent clinical examination, B-mode USG, and SWE using a Mindray DC-80 USG machine (Gurugram, Haryana, India) with a 7.5 MHz linear array probe. Lesions were evaluated based on quadrant, zone, and clock position. SWE findings were scored using the Tsukuba elasticity scoring system (grades 1-5). The Tsukuba system was classified as follows: grade 1: lesions are less less stiff than or equal to the surrounding tissue; grade 2: lesions have mixed (increased, decreased, or equal) areas of stiffness in relation to the surrounding tissues; grade 3: lesions are smaller size on the elastogram than the B-mode USG and stiffer than the surrounding tissue; grade 4: lesions are the same size on the elastogram and B-mode and are stiffer than the surrounding tissue; and grade 5: lesions are larger in size on the elastogram than the B-mode USG and are stiffer than the surrounding tissue [7].

Data analysis

The mean and standard deviation were used for the descriptive analysis of quantitative data. For categorical variables, proportion and frequency are employed. Additionally, data was shown using the proper table and pie charts.

Statistical analysis

​IBM SPSS Statistics for Windows, Version 22.0 (IBM Corp., Armonk, New York, United States), was used for data analysis. Sensitivity, specificity, positive predictive value (PPV), negative predictive value (NPV), and diagnostic accuracy of SWE were calculated and reported, along with their 95% confidence interval (CI), and compared to the gold standard HPE. Kappa statistics were used to evaluate the screening test's reliability, together with 95% CI and a p-value of <0.05. The chi-squared test was used to analyze categorical data to determine a significant relationship.

## Results

Descriptive analysis

The mean age of the study participants was 38.11±9.88 years, indicating a moderate spread around the average. The median age was 41 years, suggesting a slight skew toward older ages within the sample. The minimum and maximum ages were 18 and 66 years, respectively, reflecting a wide age range among participants. The 95% CI for the mean age ranged from 32.22 to 45.56 years, indicating that the true mean age of the population is likely to lie within this interval with 95% confidence. The descriptive analysis representation is given in Table 1.

**Table 1:** Descriptive analysis of the study population in terms of age (n=50)

Parameter	Mean±SD	Median	Min	Max	95% CI	
					Lower	Upper
Age	38.11±9.88	41	18	66	32.22	45.56

Descriptive analysis of the study population in terms of age group

The age-wise distribution of the study participants showed that the largest proportion belonged to the 30-39-year age group (34%; n=17). This was followed by the 40-49-year age group, which accounted for 28% (n=14) of the participants. Individuals aged up to 29 years constituted 18% (n=9) of the study population. Participants in the 50-59-year age group comprised 16% (n=8), while those aged 60 years and above represented the smallest group at 4% (n=2). Overall, the majority of participants were between 30 and 49 years of age, indicating a predominance of middle-aged individuals in the study. The description analysis of the age group in the study population is given in Table 2.

**Table 2:** Descriptive analysis of the study population in terms of age group (n=50)

Age group (years)	Frequency (n)	Percentage (%)
Up to 29	9	18
30-39	17	34
40-49	14	28
50-59	8	16
60 and above	2	4

Distribution of breast lesions in the study population

Right-sided involvement was the most prevalent, occurring in 48% of cases (n=24). Left-sided engagement, which made up 42% (n=21), came next. Ten percent of subjects (n=5) had bilateral participation. Right-sided cases were slightly more common than left-sided cases, with unilateral participation predominating overall. The description analysis of the distribution of breast lesions is given in Table 3.

**Table 3:** Descriptive analysis of right/left/both side lesions in the study population (n=50)

Side	Frequency (n)	Percentage (%)
Left	21	42
Right	24	48
Both	5	10

Analysis of sonoelastography in the study population

**Figure 1:**
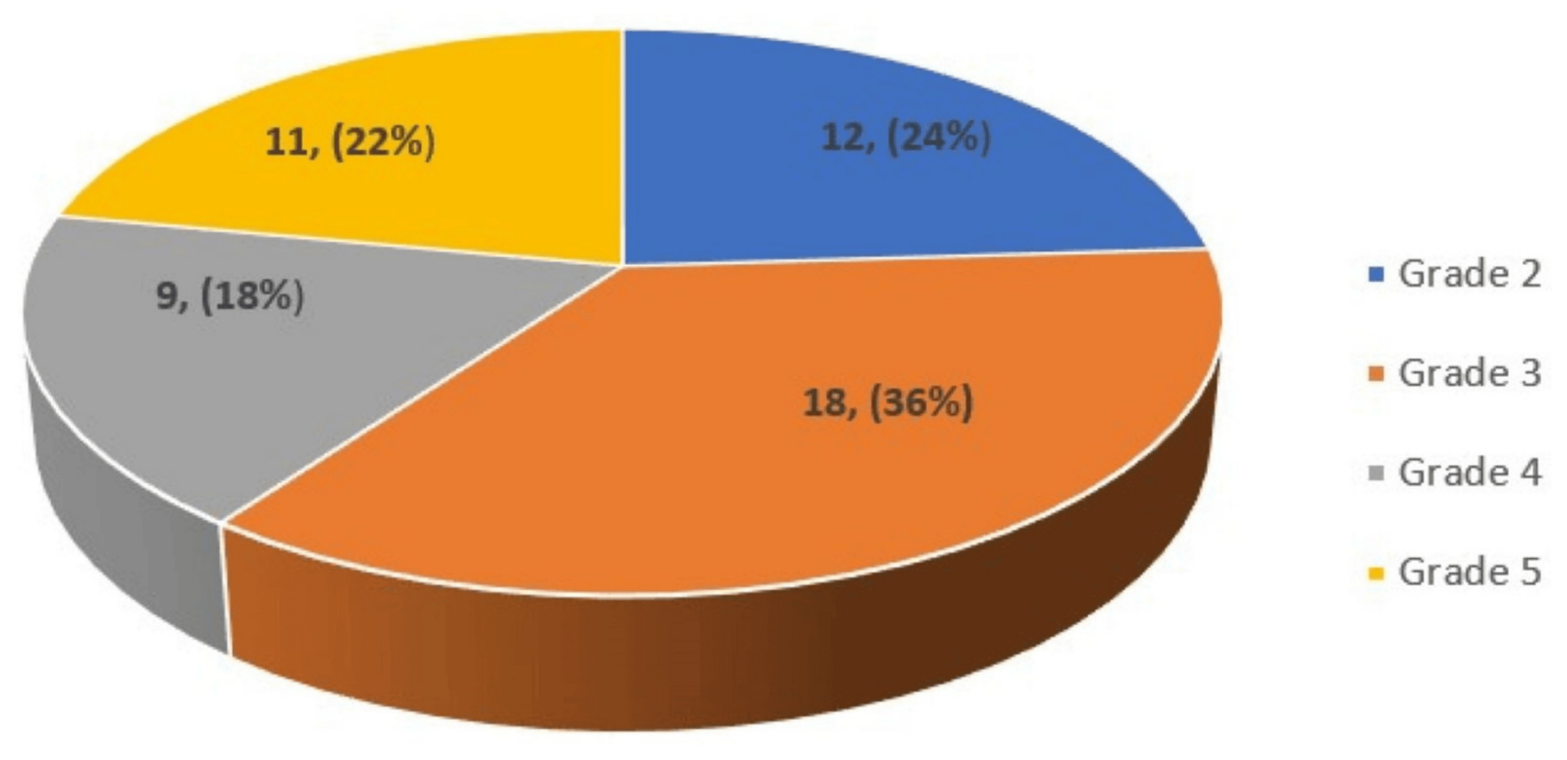
Pie diagram showing the Tsukuba elasticity grading distribution in the study population (n=50). Frequency is given in the chart and percentage is given inside the parenthesis.

The Tsukuba elasticity grading analysis study revealed that grade 3 has the highest frequency at 18, followed by grade 2, grade 5, and grade 4 with frequencies of 12, 11, and 9, respectively. The graphical representation is given in Figure 1.

Descriptive analysis of BI-RADS in the study population

**Figure 2:**
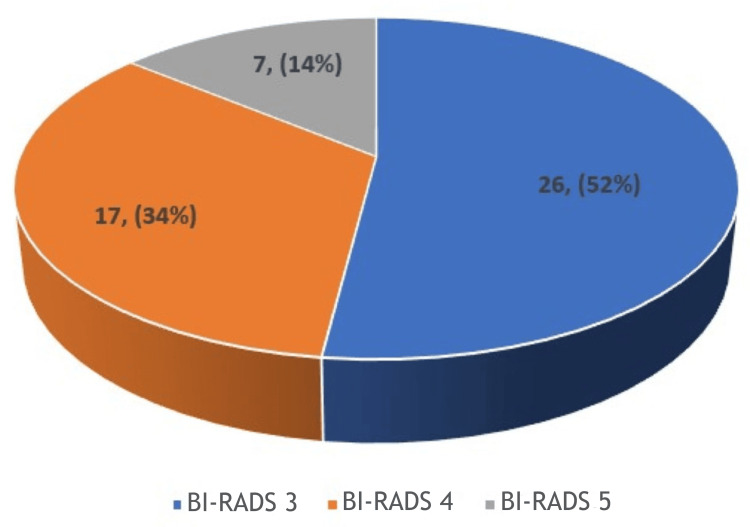
Pie diagram showing the BI-RADS category distribution in the study population (n=50). Frequency is given in the chart and percentage is given inside the parenthesis. BI-RADS: Breast Imaging-Reporting and Data System

On sonoelastography assessment, BI-RADS category 3 was the most frequently observed finding, seen in 52% of cases (n=26). This was followed by BI-RADS category 4, which accounted for 34% (n=17), while BI-RADS category 5 was the least common, observed in 14% (n=7). The graphical representation is given in Figure 2.

**Figure 3:**
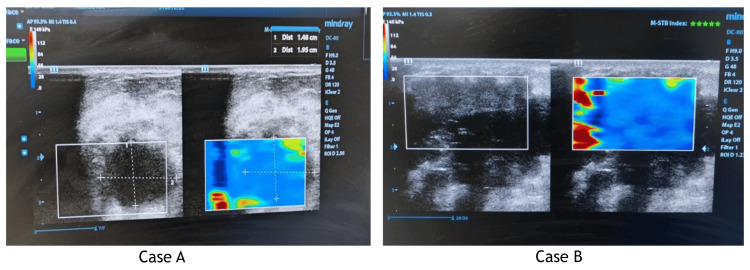
B-mode USG images of representatives cases. Case A: a 51-year-old female patient. Diagnosis: BI-RADS 4 lesion with a Tsukuba elasticity score of 4. HPE: ductal carcinoma. Case B: a 46-year-old female patient. Diagnosis: BI-RADS 4 lesion with a Tsukuba elasticity score of 4. HPE: ductal carcinoma BI-RADS: Breast Imaging-Reporting and Data System; USG: ultrasonography; HPE: histopathological examination

Figure 3 shows a heteroechoic mass lesion in two cases (Case A and Case B).

A 51-year-old woman with a painless tumor under her left nipple is shown in Case A. In the retroareolar area, B-mode USG reveals a heteroechoic mass lesion with irregular margins that may be indicative of BI-RADS 4. The entire hypoechoic lesion on the USG elastrogram is primarily blue, with a score of 4.

**Figure 4:**
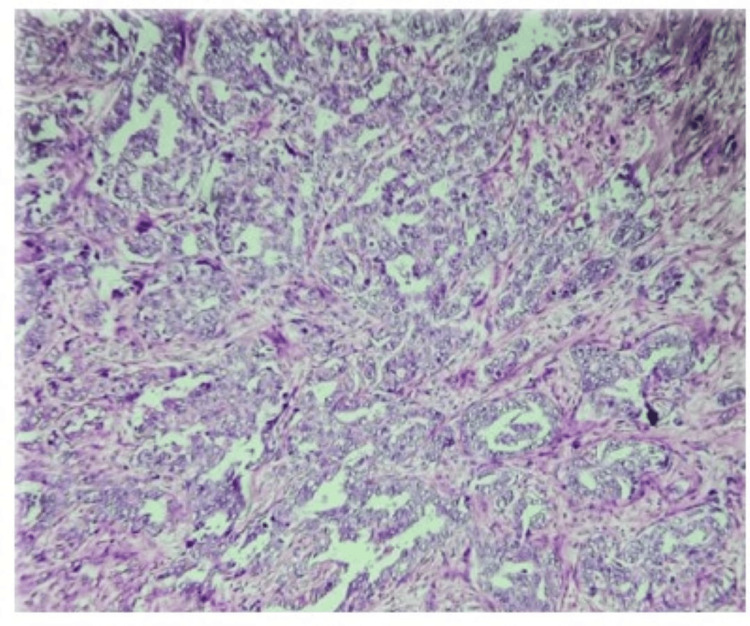
Histopathology image of Case B (a 46-year-old female patient) corresponding to Figure 3 (image on 10× magnification, H&E staining)

A 46-year-old woman in Case B complained of a painless tumor in her right breast. Heteroechoic mass lesions with irregular margins suggestive of BI-RADS 4 are seen on B-mode USG. The entire hypoechoic lesion on the USG elastrogram is primarily blue, with a score of 4. Histopathology revealed that the lesions in both cases (Case A and Case B) were ductal carcinoma. A histopathology image of Case B (a 46-year-old female patient) is shown in Figure 4. Five incidences of invasive ductal carcinoma and eight cases of DCIS were found in our study.

Descriptive analysis of the final histopathological diagnosis (HPE)

**Figure 5:**
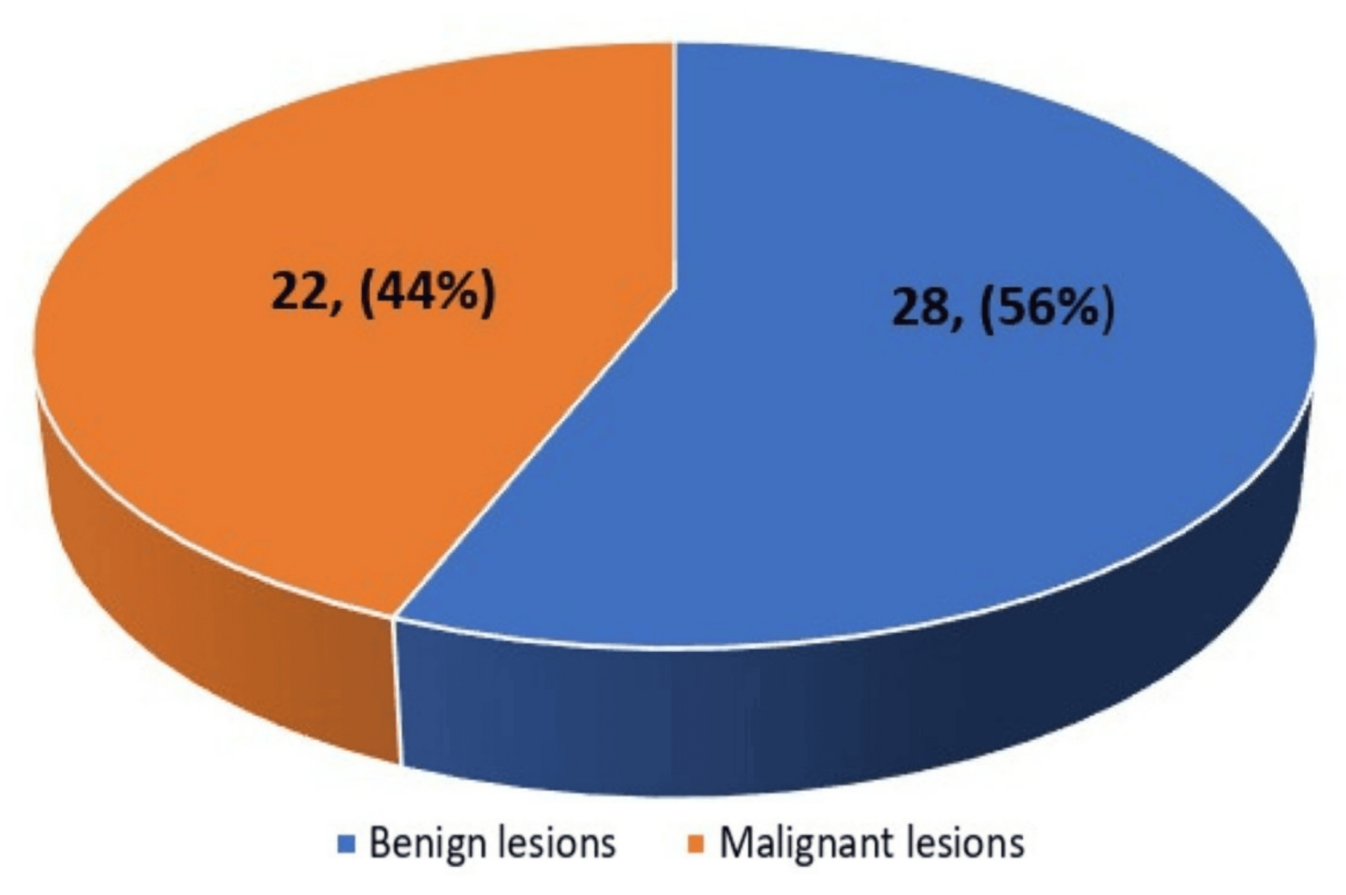
Pie diagram showing the final histopathological diagnosis (HPE) distribution in the study population (n=50). Frequency is given in the chart and percentage is given inside the parenthesis. HPE: histopathological examination

On the final HPE, benign lesions were more common, accounting for 56% of cases (n=28), while malignant lesions constituted 44% (n=22) of the study population. Overall, benign pathology predominated in the final diagnosis. The graphical representation is given in Figure 5.

In our investigation, we also discovered two phyllodes tumor instances, two cases of chronic mastitis, two cases of granulomas, one case of hemorrhagic cyst, one case of intraductal benign papilloma, and one case of fibroadenosis. These were the other benign masses that we discovered. Two cases of invasive and inflammatory lobular carcinoma and one case of medullary carcinoma were discovered among other malignant tumors.

Relationship between sonoelastography grade category and ultimate histopathology diagnosis (HPE)

The association between sonoelastography grade category and final histopathological diagnosis (HPE) was analyzed and found to be statistically significant. Among cases categorized as malignant on sonoelastography, 72.72% (n=16) were confirmed as malignant on HPE, while 14.28% (n=4) were benign. Conversely, among cases categorized as benign on sonoelastography, 85.71% (n=24) were confirmed as benign on HPE, whereas 27.27% (n=6) were malignant. The observed association was highly significant (p<0.001), indicating a strong correlation between sonoelastography grading and final histopathological diagnosis. The predictive validity analysis is shown in Table 4.

**Table 4:** Association of the final histopathological diagnosis (HPE) with sonoelastography grade category of the study population (n=50) The chi-squared test value is 15.18 with 1 degree of freedom. HPE: histopathological examination

Sonoelastography grade category	Final histopathological diagnosis (HPE)		Chi square	P-value
	Malignant	Benign		
Malignant	16 (72.72%)	4 (14.28%)	15.18 (dF=1)	<0.001
Benign	6 (27.27%)	24 (85.71%)		

Predictive validity of sonoelastography grade in comparison to the final histopathological diagnosis (HPE)

The diagnostic performance of sonoelastography was evaluated using standard validity parameters. The sensitivity of the test was 72.72% (95% CI: 54.17-91.32%), indicating a good ability to correctly identify malignant cases. The specificity was 85.71% (95% CI: 65.7-94.3%), reflecting a high accuracy in correctly identifying benign lesions. The false positive rate was 14.28% (95% CI: 4.6-23.8%), while the false negative rate was 27.27% (95% CI: 8.59-45.95%). The PPV was 80% (95% CI: 67.6-100%), suggesting that 80% of lesions classified as malignant were truly malignant on histopathology. Similarly, the NPV was 80% (95% CI: 57.5-100%), indicating reliable exclusion of malignancy in cases categorized as benign. Overall, sonoelastography demonstrated a diagnostic accuracy of 80% (95% CI: 68.9-91.1%), supporting its usefulness as a reliable adjunctive tool in differentiating benign and malignant lesions. The predictive validity analysis is shown in Table 5.

**Table 5:** Predictive validity of sonoelastography grade category compared to the final histopathological diagnosis (HPE) (n=50) HPE: histopathological examination

Parameter	Value	95% CI	
		Lower	Upper
Sensitivity	72.72%	54.17%	91.32%
Specificity	85.71%	65.7%	94.3%
False positive rate	14.28%	4.6%	23.8%
False negative rate	27.27%	8.59%	45.95%
Positive predictive value	80%	67.6%	100%
Negative predictive value	80%	57.5%	100%
Diagnostic accuracy	80%	68.9%	91.1%

## Discussion

There were 50 female cases in our study. The mean age of cases was 38.11 years with a standard deviation of 9.88. The majority of subjects belonged to the 30-39-year age group, equating to 34%, followed by the 40-49-year age group, equating to 28% of the total population.

As per studies by Sandhu et al. [14] and Somdatta and Baridalyne [15], the statistics of age in our study are in accordance with the current direction of breast cancer incidence in the Indian population, where there is an increase in the number of women between the ages of 25 and 40.

Of the diseases of the breast that have been researched, 42% of them are on the left side, 48% on the right side, and 10% on both breasts.

In our study cases, by final histopathological diagnosis (HPE), malignant lesions were 44%, while benign lesions were 56% of the total. According to an analysis by Schoonjans and Brem [16], fibroadenoma is the benign breast tumor that is most frequently diagnosed, whereas invasive ductal carcinoma is the most prevalent malignant mass. The incidence of DCIS is found to be high among malignant tumors [13]. Five incidences of invasive ductal carcinoma and eight cases of DCIS were found in our study.

According to the sonoelastography grading, 24% of the study population had grade 2 score lesions, 36% grade 3 score lesions, 18% grade 4 score lesions, and 22% grade 5 score lesions.

As regards the BI-RADS grading, of the BI-RADS categories, BI-RADS 3 is reported in 52%, BI-RADS 4 in 34%, and BI-RADS 5 in 14%.

The sensitivity and specificity of sonoelastography grading in breast lesion characterization with BI-RADS 3 and above are 72.72% (95% CI: 54.17-91.32%) and 85.71% (95% CI: 65.7-94.3%), respectively.

In breast lesion evaluation with BI-RADS 3 and above categories using sonoelastography, the PPV equates 80% (95% CI: 67.6-100%), while the NPV equates 80% (95% CI: 57.5-94.3%).

In our study group, 16 cases (72.72%) of pathologically confirmed malignancies were diagnosed accurately as malignant, while 24 cases (85.71%) of benign breast lesions were classified correctly as benign using sonoelastography grades 2 and 3.

In evaluating breast lesions with BI-RADS 3 and above using the technique of sonoelastography, the false positive rate is 14.28% (95% CI: 4.6-23.8%), while the false negative rate equates 27.27% (95% CI: 8.59-45.95%).

Sonoelastography misinterpreted four cases (14.28%) of benign breast masses as malignant, including two cases of giant fibroadenoma and two cases of fibroadenoma with an inflammatory background. Potential causes of false positive results on B-mode USG are due to fibrotic components and undetected tissue calcification.

When evaluating breast lesions with BI-RADS 3 and higher categories, sonoelastography had an overall diagnosis accuracy of 80% (95% CI: 68.9-91.1%). The comparison of the final diagnosis between sonoelastography grades and HPE is statistically significant (p<0.005).

In a research by Raza et al. [17], 84% of malignant lesions had elasticity values between 4 and 5. In our investigation, 85.71% of benign lesions had elasticity values of 2 or 3, while 72.72% of malignant masses had elasticity ratings of 4 or 5.

Sonoelastography's sensitivity and specificity varied from 67% to 83% and from 86.7% to 90%, based on studies conducted by Thomas et al. and Choi et al. [18,19]. According to these studies, elastographic results added to traditional B-mode USG can increase sensitivity and specificity.

In a comparison study between sonoelastogram and dynamic MRI for BI-RADS 3 and higher category lesions, Eisa et al. [20] found that sonoelastography showed an 84% sensitivity. The study had a specificity of 84% for sonoelastography. In our investigation, sonoelastography has a high specificity for identifying malignant breast masses when compared to earlier research.

The study is conducted in a single center with only 50 study participants; therefore, the small sample size is a limitation of the study. Validation of the study in a larger sample will help provide stronger and more reliable results.

## Conclusions

The sonoelastogram is a useful tool in predicting the nature of the breast masses. Use of sonoelastography with standard B-mode USG improves the detection of breast lesions. Sonoelastography is a useful method for assessing breast abnormalities and may help reduce unnecessary biopsies.

To conclude, USG elastography is a relatively novel imaging modality that offers speed and efficiency. A better and more focused approach and management are made possible by the incorporation of elastography onto a conventional ultrasound machine. This allows for the combination of morphological ultrasound examination of the breast and elastography assessment of the breast lesion in a single exam.
